# Daratumumab treatment for kidney-involved light chain deposition disease prevents renal function progression: a case report with 3 years of follow-up and review of the literature

**DOI:** 10.3389/fonc.2025.1466323

**Published:** 2025-01-30

**Authors:** Xueying Chen, Jie Sun, Pingyan Shen, Zijin Chen, Wen Zhang

**Affiliations:** ^1^ Department of Nephrology, School of Medicine, Ruijin Hospital Shanghai Jiao Tong University, Wuxi, Jiangsu, China; ^2^ Department of Nephrology, Ruijin Hospital, Shanghai Jiao Tong University School of Medicine, Shanghai, China

**Keywords:** light chain deposition disease, free light chain, daratumumab, renal function, proteinuria

## Abstract

Light chain deposition disease (LCDD) is a clonal plasma cell disorder characterized by the deposition of nonamyloid monoclonal light chains in multiple organs. It can affect various systems throughout the body, mainly the kidneys. Symptoms may include renal insufficiency, proteinuria, hematuria, and others. Due to the lack of effective treatment, LCDD patients with kidney involvement often progress to chronic kidney failure, ultimately requiring renal replacement therapy. Daratumumab, an anti-CD38 monoclonal antibody, is primarily used for the treatment of relapsed and refractory multiple myeloma. Recent studies have shown that daratumumab also has an encouraging effect on light-chain amyloidosis. Here, we report the case of an LCDD (κ chain) patient with proteinuria, renal insufficiency, and anemia who was followed up for 3 years, during which he received daratumumab treatment. After the daratumumab treatment, the hematologic response continued progressing to a complete response without any adverse effects and continuous renal function improvement at a low serum free light chain (sFLC) level. This case shows that daratumumab is effective at treating LCDD. For LCDD patients with kidney involvement, frequent monitoring and active control of free light chain levels are necessary, as reaching the lowest sFLC of < 20 mg/L may help to improve kidney function.

## Introduction

Monoclonal immunoglobulin deposition disease (MIDD), a rare group of disorders, include light chain deposition disease (LCDD), heavy chain deposition disease (HCDD), and mixed light and heavy chain deposition disease (LHCDD). LCDD is characterized by the deposition of nonamyloid monoclonal light chains in multiple organs. As light chains are mainly filtered by the kidneys, the kidneys are the primary organ affected by LCDD. Renal involvement in LCDD often presents with renal insufficiency, proteinuria, hematuria, and other related symptoms. The heart and liver are the most common sites of extrarenal deposition, where they may lead to heart failure and liver dysfunction, among other associated symptoms (1–3).

Treatment of LCDD is mainly based on multiple myeloma treatments, primarily proteasome inhibitors (bortezomib), immunomodulatory agents (lenalidomide and thalidomide), and autologous stem cell transplantation (ASCT). Given the rarity of LCDD and absence of randomized clinical trials, most treatment insights are derived from case reports or retrospective studies. Treatment outcomes in LCDD are generally suboptimal, with renal involvement frequently progressing to end-stage kidney disease, often necessitating renal replacement therapy. Over 50% of LCDD patients develop multiple myeloma (MM) or other lymphoproliferative disorders, and those who are also diagnosed with multiple myeloma tend to have a poorer prognosis (1, 4).

## Case report

**Figure 1:**
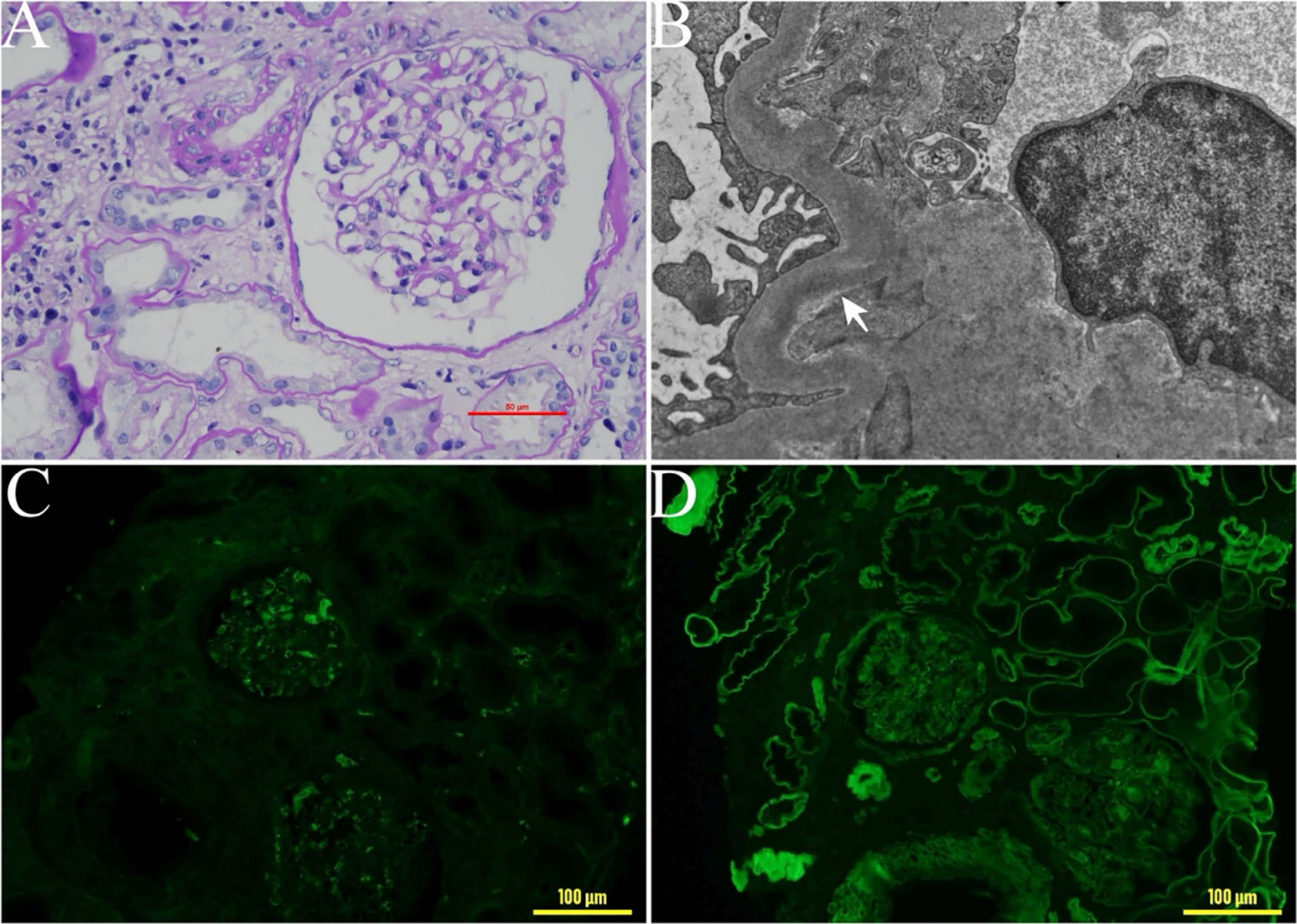
Pathological changes in renal biopsy tissue. **(A)** Light microscopy of a renal biopsy sample: significant widening of the mesangial area in the glomeruli, with increased matrix predominance and focal nodular changes. ×400. **(B)** Electron microscopy findings. Electron-dense deposits were detected along the glomerular basement membrane (arrowhead). **(C)** Immunohistochemical staining for λ light chain ×200. **(D)** Immunohistochemical staining for κ light chain, ×200.

A 47-year-old male was found to have elevated creatinine (126 µmol/L) during a routine physical in March in 2018, indicating potential renal dysfunction. However, he did not pursue further medical evaluation or follow-up at that time. In January 2020, the patient developed periorbital edema, dizziness, fatigue, and poor sleep following physical exertion. He presented to the hospital for evaluation in May 2020. Laboratory tests revealed elevated creatinine (289umol/L), a reduced estimated glomerular filtration rate (eGFR) of 21.3 ml/min/1.73 m^2^, serum free light chain (sFLC) κ 36.8mg/L, sFLC λ 44.7mg/L, a κ/λ ratio 0.823, anemia (hemoglobin 88g/L), proteinuria (quantitative urine protein 370 mg/24 h), and negative immunofixation electrophoresis result for both serum and urine. Renal pathology showed significant widening of the mesangial area in the glomeruli, with increased matrix predominance and focal nodular changes (**Figure 1A**). Congo red staining was negative. Additionally, renal tubules atrophy and interstitial fibrosis were evident. Immunofluorescence revealed of κ light chains deposition in the glomeruli, renal tubular basement membranes, and Bowman’s capsule wall (**Figures 1C, D**). Electron microscope revealed the deposition of striped electron-dense material on the inner side of the glomerular basement membrane (**Figure 1B**). Renal pathology confirmed the diagnosis of LCDD with severe tubular atrophy and interstitial fibrosis ranging from 50% to 75% of the renal tissue. To investigate potential co-occurrence with hematological diseases such as multiple myeloma, a bone marrow aspiration biopsy was performed. Bone marrow examination revealed 2% plasma cells infiltration, with a normal cytogenetic profile. Flow cytometry showed that 82.8% of plasma cells expressed CD38 and CD138. Based on these pathological findings, the patient was diagnosed with κ-LCDD. To reduce the light chain burden and preserve renal function, chemotherapy was initiated with bortezomib-based regimen, including bortezomib, dexamethasone, and cyclophosphamide), administered monthly. The patient underwent a total of six sessions of bortezomib-based chemotherapy, with the final session completed in November 2020. At that time, laboratory results showed a creatinine level of 238 μmol/L, an eGFR of 26.9 ml/min/1.73 m², urine protein of 166 mg/24h, and sFLC κ of 27.6 mg/L, with a κ/λ ratio of 1.52. Chemotherapy was subsequently discontinued. We recommended consultation with the hematology department regarding ASCT; however, the patient declined.

**Figure 2:**
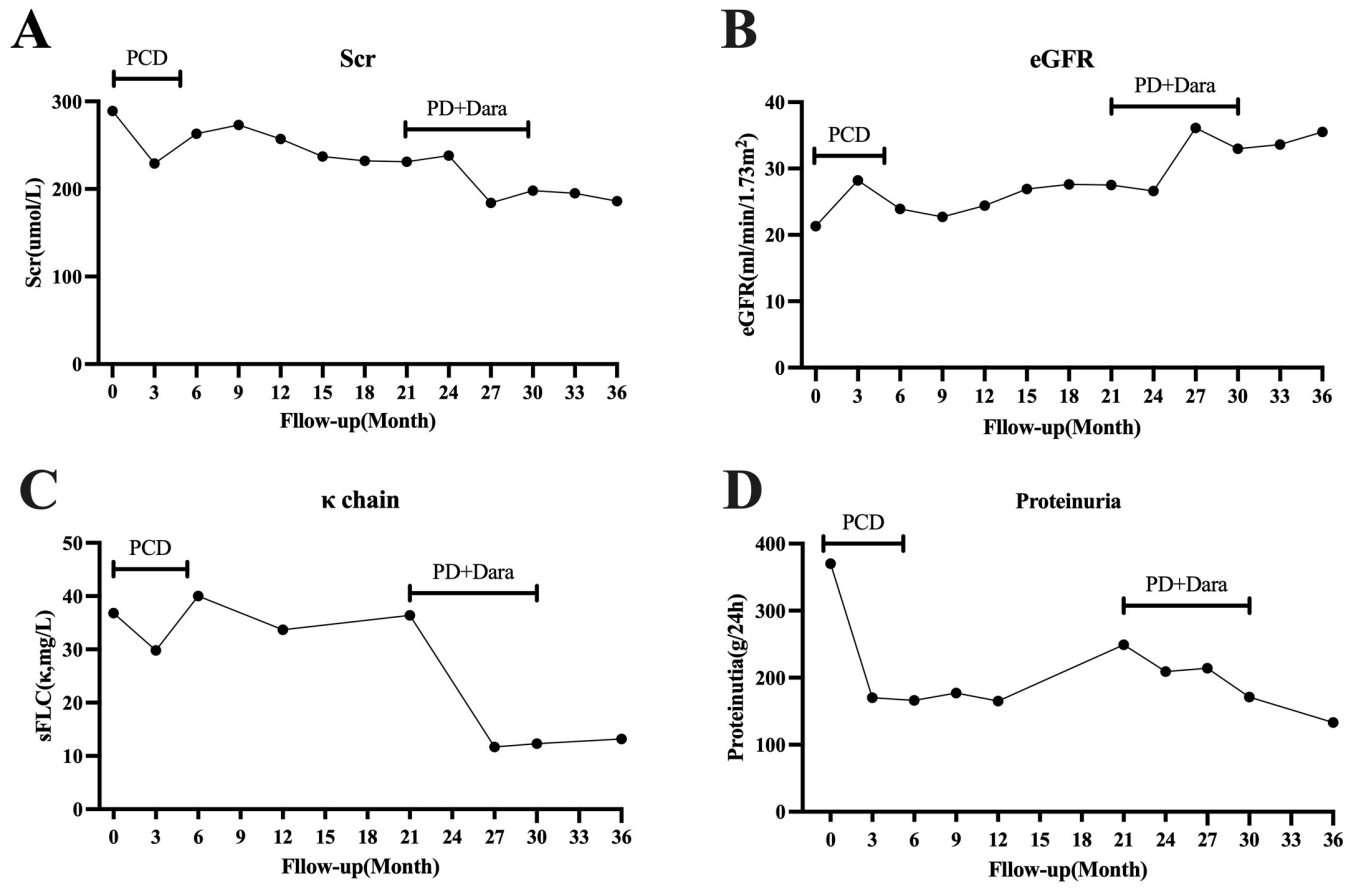
The patient’s clinical course during follow-up. **(A)** Changes in Scr levels. **(B)** Changes in eGFR. **(C)** Changes in serum free light chain κ. **(D)** Changes in proteinuria. PCD, bortezomib, dexamethasone, and cyclophosphamide; PD, bortezomib and dexamethasone; Dara, daratumumab. Scr, serum creatinine; eGFR, estimated glomerular filtration rate; sFLC, serum free light chain.

At the February 2022 follow-up, the sFLC κ was 36.4 mg/L, sFLC λ was 35.8 mg/L, κ/λ ratio 1.02, representing a significant increase compared to the previous result. Progressive disease was suspected. Considering the significantly increased percentage of CD38CD138 plasma cells detected by flow cytometry in the bone marrow prior to treatment, as well as the presence of CD38 plasma cells in the renal pathology, we decided to initiate a bortezomib-based regimen. This included bortezomib 1.3 mg/m² by subcutaneous injection on days 1 and 8, once a month, combined with dexamethasone 20 mg on the same days. Additionally, daratumumab was administered at a dose of 16 mg/kg on days 1 and 8, once a month. A total of eight treatment cycles were completed, with the final cycle concluding in December 2022. Following treatment, the patient’s renal function improved, and sFLC decreased to 11 mg/L and remained at low levels. After discontinuation of treatment for 6 months, a June 2023 follow-up examination revealed the following results: creatinine 186 μmol/L, eGFR 35.5 ml/min/1.73 m2, sFLC κ 13.2 mg/L, sFLC λ was 16.0 mg/L, κ/λ ratio 0.83, and urine protein 133 mg/24 h (**Figure 2**). The patient’s condition was stable, and there was no sign of progressive disease. The patient did not experience any significant adverse reactions during the treatment process.

## Discussion

This article reports a case of LCDD in which kidney involvement was the primary clinical manifestation, with a diagnosis at the young age of 47. According to the literature, the median age of onset for LCDD is 55-64 years, with a male-to-female ratio of approximately 2:1. κ light chain deposition is the predominant type of LCDD (2, 3, 5). Since light chains in the blood are mainly filtered by the glomeruli of the kidneys, the kidneys are the primary organs affected by light chain deposition, most extrarenal deposits being found in the heart and liver (1–3). Almost all LCDD patients with kidney involvement are diagnosed with renal dysfunction, 30-64.2% of whom are diagnosed with CKD at stages 4-5 (2, 3), and approximately 16-22.9% of these patients have already started renal replacement therapy at the time of diagnosis (3, 5, 6). Proteinuria is also one of the main clinical features of kidney involvement in LCDD, with 36-53% of patients having proteinuria in the nephrotic range and 8.6-29% presenting with nephrotic syndrome (1, 3, 5). High blood pressure and hematuria are common clinical manifestations of kidney-involved LCDD (2, 3, 5, 6). The primary manifestation of kidney involvement in LCDD is glomerular damage, characterized by nodular glomerular sclerosis on pathology (1–3). However, some patients exhibit tubulointerstitial damage and only present with mild proteinuria (7). In the case reported here, the patient primarily presented with renal dysfunction, with a creatinine level of 289 µmol/L, an eGFR of 21.3 mL/min/1.73 m^2^, stage 4 CKD, and only mild proteinuria at 370 mg/24 hours. Pathological examination suggested LCDD with significant tubular atrophy and interstitial fibrosis.

The treatment of LCDD often draws reference from plasma cell disorders such as multiple myeloma and amyloidosis. Due to the rarity of LCDD, clinical experience is mostly derived from case reports or small-scale retrospective studies [10]. The primary goal of LCDD treatment is to reduce the production and tissue deposition of light chains, thereby preventing further damage to organ function. Commonly used medications include proteasome inhibitors (bortezomib) and immunomodulators (lenalidomide, thalidomide), among others. Bortezomib is a proteasome inhibitor that plays a significant role in the treatment of multiple myeloma and amyloidosis. The mechanism of kidney injury in LCDD is closely related to the activation of nuclear factor κB (NF-κB) in proximal tubular cells and mesangial cells in the glomerulus. Bortezomib can prevent glomerular sclerosis and improve kidney function by inhibiting NF-κB (4, 8). In 2009, bortezomib was first reported for the treatment of LCDD, for which it achieved favorable hematological remission and renal responses (9). Since then, there have been some reports on the use of bortezomib for LCDD (10, 11), showing good efficacy and safety. The overall complete response rate with bortezomib-based chemotherapy regimens is 55.6% (12).

ASCT is an effective treatment method for hematological diseases, also plays an important role in the treatment of LCDD patients. Compared to other treatment methods, ASCT has a higher hematologic remission rate (3, 13–15). The renal response in patients with MIDD is closely associated with the hematological response. Among patients achieving a hematological response of a complete response (CR) or very good partial response (VGPR), the renal response rate is 57%, compared to 33% in those with partial response (PR) or no response (NR) (16). Sayed RH et al. (3) reported on 16 patients with LCDD who underwent melphalan-conditioned ASCT. Among the 11 patients who were not on renal replacement therapy (RRT) prior to ASCT, the median GFR improved from 24 to 38 mL/min/1.73m² at follow-up. Of the survivors, 13 achieved CR, and 2 PR. Notably, only 3 patients with CKD stage 4 at the time of ASCT required dialysis later.

Patients with LCDD often progress to chronic renal failure and eventually require renal replacement therapy. Previous studies have shown that kidney transplantation in LCDD patients without achieving hematologic response or effective control of light chain production results in poor prognosis. After transplantation, 71% (5/7) of patients relapse, with a median time to relapse of 33.3 months, and a median time to progression to ESRD after relapse of 10.9 months (17). Sayed RH et al. reported 4 patients who underwent ASCT either during or after renal transplantation. None of these patients showed evidence of LCDD relapse during postoperative follow-up (3).

CD38 is a type II transmembrane glycoprotein that is widely expressed on various blood cells. It is involved in receptor-mediated adhesion and leukocyte rolling. Malignant cells, particularly plasma cells in multiple myeloma, often exhibit elevated expression of CD38 (18). Daratumumab is the first approved humanized anti-CD38 monoclonal antibody. It directly binds to CD38 and exerts its anti-tumor effects through multiple mechanisms, including antibody-dependent cellular cytotoxicity (ADCC), antibody-dependent cellular phagocytosis (ADCP), direct induction of cellular apoptosis, complement-dependent cytotoxicity (CDC), and modulation of extracellular ectoenzyme activity (19). It is mainly used for the treatment of relapsed and refractory multiple myeloma. Recent research has shown that daratumumab also has good efficacy in treating light chain amyloidosis, as it offers better hematological response and organ response rates than traditional treatment approaches (20, 21).

In 2020, daratumumab was first reported as a treatment for LCDD patients. A study of eight LCDD patients who had previously undergone treatments such as proteasome inhibitors and immunomodulators showed disease progression (all patients had a baseline bone marrow plasma cell infiltrate >10%). Daratumumab was administered as a treatment. Following treatment, seven of the patients achieved a PR, and four of them had VGPR. Among the treated patients, 85.7% (6 out of 7) experienced stable kidney function, and one patient showed a significant improvement in kidney function, their eGFR increasing from 30 mL/min/1.73 m^2^ before treatment to 45 mL/min/1.73 m^2^ (22). Another study reported six LCDD patients who had not achieved complete remission (CR) after first-line chemotherapy. These patients received consolidation treatment with daratumumab for four cycles. One patient improved from PR to CR, three patients achieved VGPR, and 50% of the patients had their serum free light chain ratios return to normal after treatment (18).

After diagnosis, the patient received chemotherapy based on bortezomib. Following six cycles, a follow-up examination showed a reduction in sFLC levels and achievement hematologic remission. Chemotherapy was paused, and the patient declined further treatment with ASCT. Regular monitoring of serum free light chains, kidney function, and proteinuria was continued. One year after suspending chemotherapy, evaluation showed a significant increase in the absolute value of sFLC κ and proteinuria, raising concern for disease progression. Although the patient achieved hematologic remission with bortezomib-based treatment, there was no significant organ response, and serum creatinine levels did not decrease further. Daratumumab has shown improved hematologic and organ responses in patients with amyloidosis. To improve the patient’s prognosis, treatment was resumed with bortezomib-based regimens in combination with daratumumab. After eight cycles of treatment, the patient’s kidney function and proteinuria improved further. Serum free light chain κ levels decreased compared to those before treatment. After discontinuing treatment for 6 months, the patient’s eGFR increased from 27.5 mL/min/1.73 m2 before treatment to 35.5 mL/min/1.73 m^2^. Urine protein decreased from 249 mg/24 h to 133 mg/24 h, and sFLC κ decreased from 36.4 mg/L to 13.2 mg/L.

The most distinctive feature of our case is that following daratumumab treatment, both sFLC level and renal function improved. Remarkably, six months after chemotherapy, both sFLC and renal function remained stable. This finding prompts clinical consideration: Do patients with LCDD affecting renal function require more aggressive treatment to achieve lower target sFLC levels? Research has shown that in patients with amyloidosis, a lower sFLC at the end of treatment helps to improve survival rates and organ response. Achieving an involved free light chain (iFLC) (involved in FLC) ≤ 20 mg/L at the end of treatment is associated with higher organ response rates and improved hematological progression-free survival and overall survival (OS) (23). Another study also found that patients with posttreatment iFLC less than 10 mg/L had significantly better survival rates and organ responses than patients with higher iFLC (24). Our patient’s iFLC dropped to a low of 27.6 mg/L during treatment with bortezomib and rapidly increased after stopping treatment. During follow-up, an increase in sFLC was noted. Although the patient had achieved hematologic remission with bortezomib-based treatment, renal function showed limited improvement. As a result, we decided to initiate treatment with daratumumab in combination with bortezomib. During the treatment, the patient’s iFLC dropped to a low of 11 mg/L and remained low, being 13.2 mg/L at the re-examination 6 months after stopping treatment.

## Conclusion

Daratumumab is an effective drug for treating LCDD, and its early application in this disease may lead to further improvements in patient renal function and prognosis. Furthermore, we believe that reducing the target sFLC as much as possible will help to further improve patient target organ function and prognosis, though further research is needed to explore this possibility.

## Data Availability

The raw data supporting the conclusions of this article will be made available by the authors, without undue reservation.
